# Significant Delay of Lethal Outcome in Cancer Patients Due to Peroral Administration of Bacillus oligonitrophilus KU-1

**DOI:** 10.1100/tsw.2006.347

**Published:** 2006-07-08

**Authors:** Sergey V. Malkov, Vladimir V. Markelov, Gleb Y. Polozov, Boris I. Barabanschikov, Alexander Y. Kozhevnikov, Maxim V. Trushin

**Affiliations:** ^1^ Kazan State University, Department of Genetics, Kremlyovskaya str. 18, 420008 Kazan, Russia; ^2^ Kazan Rehabilitation Medical Health Center "Sanatorium Krutushka", 420008 Kazan, Russia; ^3^ Institute of Biochemistry and Biophysics, PO Box 30, 420111, Kazan, Russia

**Keywords:** cancer, anticancer drug, metastasis, survival rate, Russia

## Abstract

Treatment of cancer patients remains a serious medical problem and the development of alternative treatment strategies is therefore of great importance. In this connection, we developed a new bacterial-based, anticancer method. Ten cancer patients (three males, seven females) were involved in this study. Bacterial suspension of stationary phase *Bacillus oligonitrophilus* KU-1 was used as a remedy for peroral administration. In five patients, side effects (sicchasia, slight blood, and intracranial pressure gain) were detected, but all patients showed significant delay of lethal outcome without serious side effects. In conclusion, the suggested method was, in our opinion, a good alternative to conventional chemo- and radiotherapy techniques. In order to evaluate its efficiency for various tumors, a double-blind, placebo-controlled, multicenter study is needed.

## INTRODUCTION

Oncological diseases remain a significant health problem despite enormous scientific efforts and investments during the last decades. Numerous protocols for chemo- and radiotherapeutic treatment of various cancers have been developed[1,2,3,4,5,6]. However, a very low efficiency, especially in advanced cancer patients, as well as serious side effects[7,8] evoke a necessity to work up some alternative treatment strategies.

Using some theoretical backgrounds suggested by Tokin[9] and Voronkov et al.[10], a new bacterial-based, anticancer treatment strategy has been suggested. As our experience showed[11], safety and relative efficiency were the main hallmarks of the developed method.

## METHODS

Ten cancer patients (three males, seven females, age 42–76 years) were involved in this study. Although type of cancer varied from patient to patient, all of them were characterized by the presence of distant metastasis. Bacterial suspension of *Bacillus oligonitrophilus* KU-1 strain was used as remedy. The bacterial strain was isolated from the soil of Kazan city in Russia, according to published techniques[12]. After identification, *B. oligonitrophilus* KU-1 was cultivated in liquid medium of Alexandrov as described previously[11]. It has been shown that *B. oligonitrophilus* KU-1 does not have any toxicity[11]. Stationary phase *B. oligonitrophilus* KU-1 culture ($0.5–1.0 \times 10^9$ cells/ml) was used for peroral administration according to previously reported scheme[11]. It is important to note here that freshly frozen bacterial culture can be used for administration. For this purpose, a small aliquot of liquid bacterial culture (e.g., 100 ml) can be frozen in a plastic container and then unfrozen before administration. *B. oligonitrophilus* KU-1 culture can be stored in frozen form for a month without deprivation of anticancer activity. To compare difference in overall survival, we used paired Student *t* test. A $p$ value of $<0.01$ was considered to indicate significance.

## RESULTS

**Case 1** — Female, born in 1929. In 1996, sigmoid colon tumor was diagnosed. Operation on primary tumor ablation was made at Tatarstan Republican Hospital (Kazan, Russia). Two years later, tumor relapse with metastases into left ureter (left kidney was nonfunctional) and loops of thin bowels was found. Concomitant diseases were hypertension, diabetes, adiposity, and postoperative ventral hernia. In December 1998, enterostomy, ileotransversostomy, and herniotomy were performed. At bifurcation of aorta, a tumor (15 × 9 cm) was observed. Metastases into loops of small intestine and ileum were the reason for intestinal obstruction. There were carcinomatous nodes at transversal colon and small intestine. The patient was declared inoperable. The patient refused chemotherapy. Since August 1999, minimal doses of *B. oligonitrophilus* KU-1 culture were received (10 ml/day). Then, administration of bacteria increased up to 50 ml/day, but in September 2000, administration of bacteria stopped due to intolerance. In November 2000, the patient died due to stroke.

**Case 2** — Female, born in 1930. In early 1997, breast adenocarcinoma with vertebral column metastases, metastases in ribs and upper extremities determined ($T_3N_1M_1$). The associated illnesses were diabetes, allergy, and renal calcinosis. Operation on primary tumor ablation was made at All-Russian Oncological Center (Moscow, Russia). In 1997, chemotherapy and radiotherapy were performed. Since March of 1997, the patient received *B. oligonitrophilus* KU-1 culture (400 ml/day). In July 1997, bandilization of metastases was observed. After that time, bacteria administration was stopped. In autumn of 1997, bandle state was watched. However, the administration of stimulator of bone stock growth ("Bonephos") resulted in tumor relapse and death in November 1998.

**Case 3** — Female, born in 1957. In late 1999, ovary tumor with metastases into liver and abdominal cavity as well as undifferentiated rectum tumor ($T_4N_xM_1$). In February 2000, hysterectomy and resection of greater omentum were made at Government State Service "Oncology" (Kazan, Russia). Sigmostoma and rectum were not ablated. There was about 3 l of ascitic fluid in the abdominal cavity. Right ovary was in the form of thick-walled cyst (20 × 18 × 15 cm). In the liver, there were three roundish formations (up to 3 cm in diameter). In the rectosigmoid section, there was Schnitzler's metastasis (2 × 3 × 1 cm). Urine analysis (May 5, 2000): weight, 1.010; pH 6.0; glucose and protein were absent. Ultrasonography (March 31,

2000): liver was heterogeneous parenchyma near the edge of costal margin. There were hyperechoic formations (in SVI, 55 mm in diameter; in SVII, 41 mm in diameter). There were foci of disintegration in the center of each hyperechoic formation. In the left lobe of liver, there was hyperechoic formation (31 mm in diameter). Gall bladder was increased. Ultrasonography (May 6, 2000): liver was with variety of hyperechoic metastatic formations of 69 mm in diameter (some of them were with fluid inclusions). Kidneys, spleen, heart, lungs, and pancreas were without pathology according to X-ray examination. In May 2000, administration of *B. oligonitrophilus* KU-1 culture was started. The initial level of bacteria administration was 200 ml/day; since August and October, daily administration was increased up to 400 and 600 ml, respectively. Simultaneously, topotecan was received. Ultrasonography of liver (February 2, 2001): numerous metastases (47–123 mm in diameter) were detected. During all this time, the patient received daily 500–700 ml of *B. oligonitrophilus* KU-1 culture. Due to liver failure, the patient died in July 2001.

**Case 4** — Male, born in 1955. In May 2000, fiberoptic colonoscopy was made and rectosigmoid adenocarcinoma with metastases into liver revealed ($T_3N_1M_1$). Analysis of feces (May 19, 2000): color, red; stool consistency, sleek; concealed blood was present. Ultrasonography of abdominal cavity (May 18, 2000): liver – right lobe, 138 mm; left lobe, 83 mm. There was pronounced fatty infiltration. Gall bladder was contracted (49 × 21 mm) with heterogeneous contents. Pancreas – bulb, 32 mm; body, 15 mm; tail, 17 mm. Spleen was without pathology. In June 2000, rectum resection and sigmostoma performed at Samara Oncology Center (Samara, Russia). During the operation, three metastatic niduses were observed (2–3 mm in diameter). The patient rejected chemotherapy. In August 2000, administration of *B. oligonitrophilus* KU-1 culture (200–300 ml/day) was started. Ultrasonography (September 2000): there were increased echogenicity and heterogeneous parenchyma. Periportal image was slowed. Diaphragm and hepatic veins were illegible. There was acoustic shadow in the projection of gall bladder. Parenchyma of pancreas was heterogeneous with increased echogenicity. Spleen was without pathology. Liver was increased with heterogeneous structure. In V and VII segments, there were two hypoechoic structures (16 × 14 and 13 × 10 mm). Magnetic resonance imaging (MRI) procedure (January 2001): metastases were revealed in the liver (in VI segment, 8.4 × 7.8 and 14.6 × 15.2 mm; in VIII segment, 9.2 × 10.1 mm; at the border of VI and VII segments, 8.4 × 6.1 mm). Diameter of portal vein was 10.8 mm. Cholelithiasis and chronic cholecystitis were also revealed. MRI procedure (April 2001): negative dynamics observed; liver metastases increased 12.1 × 9.1 to 18.6 × 13.1 mm. Additional metastases in V segment was determined. MRI procedure (October 2001): liver was increased. Metastatic niduses were increased (22.8 × 19.5 mm in VII segment; 19.1 × 15.9 mm in VI segment). Additional metastases were revealed in VI segment (up to 12.1 mm in diameter), VII segment (up to 13.3 mm in diameter), and in II segment (up to 11.1 mm in diameter). Diameter of portal vein was 11.0 mm. There was conglomeration of glands of increased size (4.1 × 3.8 × 4.5 cm) at liver hilus. Since that time, administration of bacteria was increased up to 400 ml/day. MRI procedure (August 2002): numerous metastases were revealed in the projection of hepatoduodenal ligament. Metastases in liver were without negative dynamics. In third segment of liver, additional metastases were determined (37.6 mm in diameter). Roentgenography (July 2002): in the left lobe of lungs, metastases were revealed (1–3 cm in diameter). Ultrasonography (March 2003): in perineum, metastases (14 × 18 mm) were revealed. MRI procedure (April 2003): in the projection of rectum stump, tumor relapse was determined (metastasis about 66 × 40 mm). Since May 2003, state worsening observed (daily administration of bacteria was 200–250 ml). Due to pulmonary edema and stroke, the patient died in September 2003.

**Case 5** — Female, born in 1939. In January 1999, gently differentiated tumor of maxillary sinus was revealed ($T_3N_0M_0$). Chemotherapy (platinum-containing drugs) and radiotherapy (gamma irradiation, 90 Gy) resulted in deterioration of health state. MRI procedure (March 1999): mucous tunic was nonuniformly incrassate (from 2–5 mm); there were postoperative alterations of left maxillary sinus without tumor relapse. MRI procedure (July 1999): mucous tunic was increased up to 15 mm from the left side; tumor relapse was diagnosed. In November 1999, the patient rejected chemo- and radiotherapy and began to get *B. oligonitrophilus* KU-1 culture (300–400 ml/day). Radiodiagnostics (December 1999): there was destruction of left inner wall of maxillary sinus. From the right side, there was significant thickening of mucous tunic of maxillary sinus. In February 2000, the patient received sulfanilamides during 14-day period because of tracheitis. MRI procedure (April 2000): from the left side, there was soft formation with homogeneous structure (15–17 mm in diameter); residual tumor without negative dynamics. Due to increase of blood pressure, in autumn 2000, administration of bacteria was stopped. MRI procedure (December 2000): the previously determined soft formation was of the same sizes (without dynamics at all). During spring 2001, there was preventive administration of *B. oligonitrophilus* KU-1. MRI procedure (November 2001): in projection of the left maxillary sinus, there was soft formation (15.7 × 16.7 × 17.1 mm) without dynamics. Since November 2001, administration of bacteria (50 ml/day) was recommenced. At the end of autumn, pain strengthening was observed. In March 2002, tumor formation was ablated from the left maxillary sinus. It was found histologically that tumor was undifferentiated. Metastasis into eye-socket was revealed. In September 2002, tumor was reoperated. Since March 2002 up to December 2002, administration of bacteria was absent and patient received platinum-based drugs. Since December 2002, administration of bacteria was renewed (300 ml/day). Chemotherapy was contraindicated. MRI procedure (January 2003): pathology of brain structures was not revealed. In February 2003, last operation was made (tumor ablation). In June 2003, metastases into parotid gland were determined. In November 2003, there was a drastic worsening of health state: metastases were revealed in lower and upper jaws as well in the region of carotid artery. In January 2004, the patient died.

**Case 6** — Male, born in 1925. In April 2001, computed X-ray imaging performed and metastatic backbone tumor revealed (Fig. 1). The patient rejected chemo- and radiotherapy. Urine analysis (the same date): protein and glucose were absent; specific gravity, 1.002; reaction was acid. Since April 2001, the patient began to receive *B. oligonitrophilus* KU-1 culture (200 ml/day). Since December 2001, daily administration decreased up to 100 ml. In August 2002, total destruction of D11 and D12 bodies and significant abnormalities in D8–D10 were revealed in roentgenograph. Diagnosis: metastases in D11–D12. In July–August 2004, ultrasonography of liver, kidneys, pancreas, and spleen without pathology. In February 2004, the patient stopped administration of *B. oligonitrophilus* KU-1 culture considering him cured. In July 2004, computed X-ray imaging was made and cancer bandilization was observed (Fig. 2). The patient died on April 4, 2006, without cancer relapse.

**Case 7** — Female, born in 1935. In autumn 2003, gently differentiated stomach adenocarcinoma was revealed ($T_4N_xM_0$). After that, subtotal resection of stomach was made at Government State Service “Oncology” (Kazan, Russia). In 2004, metastases were determined in the abdominal cavity. Chemotherapy was contraindicated. Since June 2004, administration of *B. oligonitrophilus* KU-1 was begun (200 ml/day). Due to blood pressure increase, administration of bacteria was stopped in November 2004. Ultrasonography of abdominal cavity (December 2004): a gall bladder had size 81 × 32 mm and there was incrassate wall (4 mm). Contents with many concretions, anhepatic ducts were dilated. Pancreas: there were regular contours; sizes: bulb, 26 mm; body, 15 mm; tail, 22 mm. Diameter of *V. porta*, 9 mm; *V. lienalis*, 4.5 mm. Spleen (120 ×

53 mm) was with fibrous inclusions. Calculous cholecystitis was diagnosed. Since December 2004, administration of bacteria was renewed (200 ml/day). Ultrasonography (April 27, 2005): multiple metastases (35 × 39 mm in diameter at the liver hilus, 29 × 33 mm in S7–S8 segments) in liver were revealed. In the gastric remnant, tumor relapse (46 × 41 × 50 mm) was observed. The patient died in August 2005.

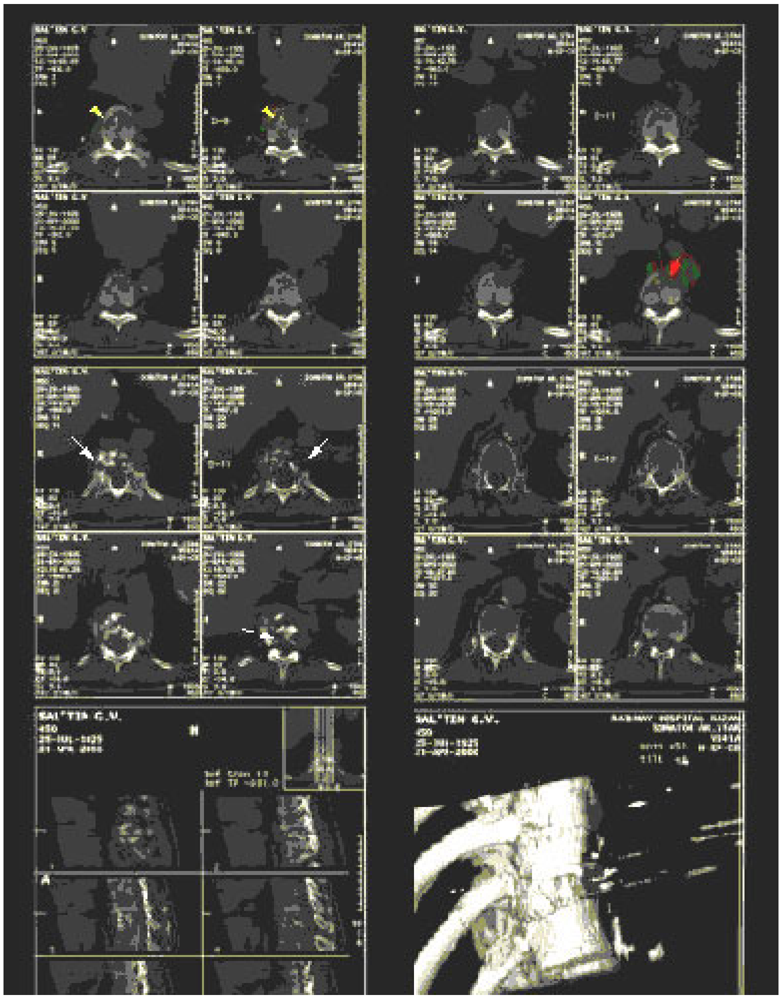

**FIGURE 1**. MRIs of patient with backbone metastases (before treatment with bacteria). Compression fracture of the Th.XI. vertebral body with wedge-shaped deformity and slight consequent kyphosis are detected. There are sclerotic bony fragments, but the presence of lytic process is also evident; this is most conspicuous in the vertebral arches. Surrounding soft tissues are somewhat widened. There is another lytic area (approximately 1.5 × 2.5 cm) on the left anterior aspect of Th.X. vertebral body adjacent to Th.X. intervertebral space, affecting the cortical bone as well. A third lytic area is demonstrated in Th.IX. Vertebral body on the right side with a size of approximately 1 × 2.5 cm. It has lobulated contours with sclerotic margins. There are moderate sclerotic degenerative appositions at the Th.XI. facet joints. There is no significant spinal canal stenosis at this point. Lytic areas in Th.XI. vertebra, involving the arches: white arrows; lytic lesion in Th.IX. vertebral body with sclerotic margin: short yellow arrow; lesion in Th.X. vertebral body at its lower rim: red arrow.

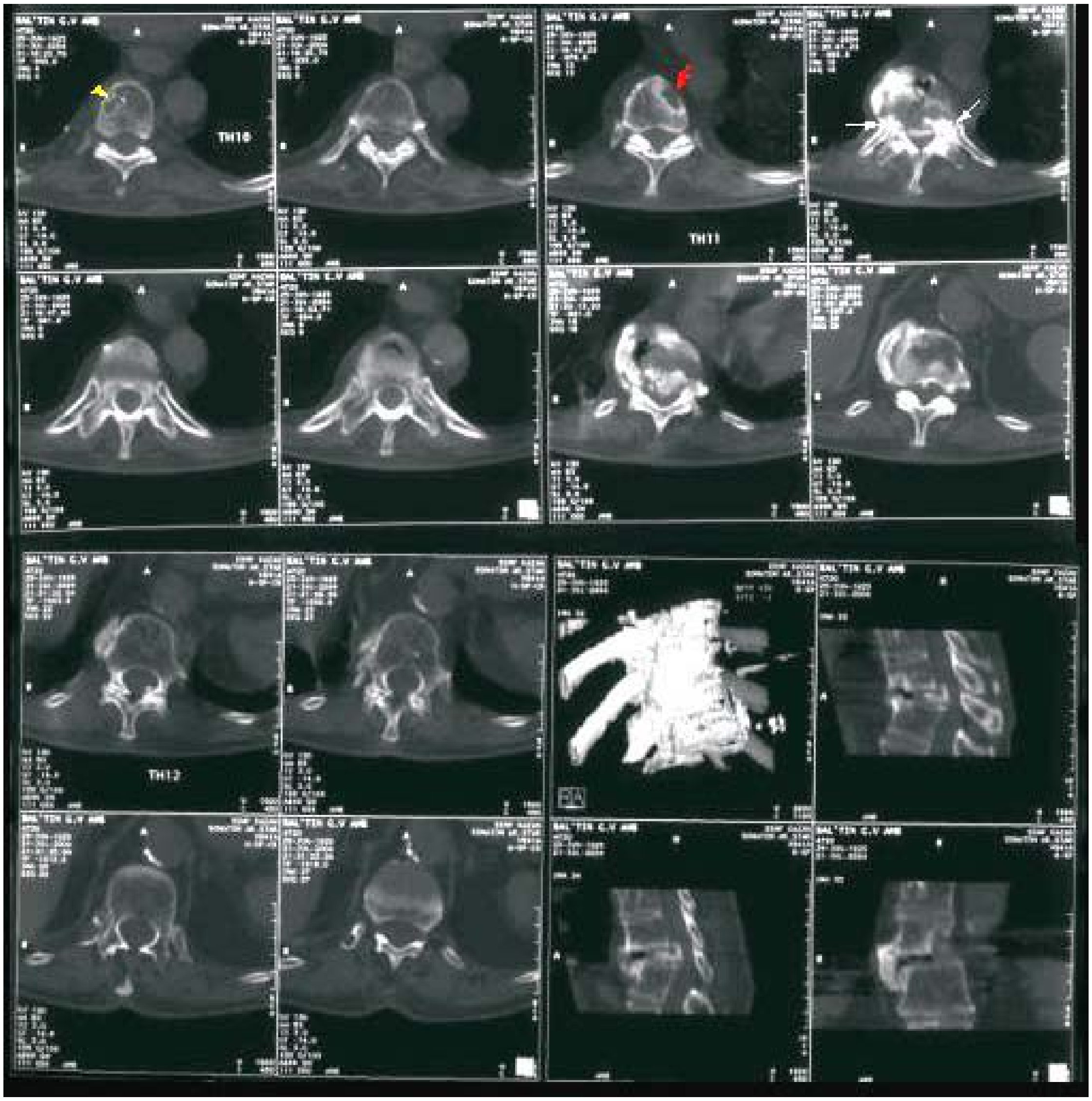

**FIGURE 2**. MRIs of patient with backbone metastases (after treatment with bacteria). There is progressive spinal deformity: collapse of the anterior part of Th.XI. vertebral body is complete with more prominent wedge-shaped deformity. Posterior sclerotic bony elements shifting towards the canal cause significant stenosis (at least 50% in AP direction). Remarkable osteophytic appositions have developed on the right lateral aspect of Th.X.–XI. intervertebral spaces. There is marked progression of sclerotic degenerative changes in the facet joints as well. There is sclerosis in the vertebral arches instead of the formerly observed lytic areas. No evidence of soft-tissue mass. The lytic area affecting the left anterior aspect of Th.X. vertebral body became demarcated by irregular slerotic margin. The lesion in the Th.IX. vertebral body is unchanged. Compression fracture of Th.XI. vertebral body was probably caused by lytic bone pathology, which seemed to affect Th.X. vertebral body as well. From the available data, the nature and dignity of this process cannot be determined with confidence; both malignant neoplasia (metastasis) and benign tumors/tumor-like lesions may be taken into consideration. The follow-up, however, reveals progression only in the secondary deformity and the accompanying degenerative changes with consolidation and demarcation of the lytic components. This could be explained by an effect of successful antineoplastic, bacterial-based treatment of a malignant tumor. The probability of malignancy would set lower. The lesion in the Th.IX vertebral body had slightly different imaging characteristics that have not changed in the follow-up period, hence it may represent benign pathology different from the one affecting Th.X–XI. segments. The most striking finding of the follow-up scan is the evolution of significant bony spinal canal stenosis. Sclerosis in place of former lytic areas in Th.XI. vertebra: white arrows; unchanged lytic lesion in Th.IX. vertebral body with sclerotic margin: short yellow arrow; lesion in Th.X. vertebral body at its lower rim with sclerotic margins: red arrow.

**Case 8** — Female, born in 1962. In August 2002, double-sided ovary cystadenocarcinoma (4 × 4 and 5 × 7 cm) with a wide distribution into peritoneum was determined ($T_4N_xM_1$). In addition, multiple metastases into greater omentum were observed. Cystatin was received before operation. In that period, the patient was very ill. In August 2002, hysterectomy and ablation of greater omentum were made at Samara Oncology Center (Samara, Russia). Since September 2002 until February 2003, the patient received *B. oligonitrophilus* KU-1 (300 ml/day). Increase in intracranial pressure was observed while blood pressure was normal. After CA125 normalization, bacteria were received with prophylactic aim (50 ml/day, a week of administration was changed by a week of interruption). In May 2004, ultrasonography revealed metastases into cellular tissue of pelvis minor. Chemotherapeutic course of treatment (cystatin, 110 mg) was perceived very badly (there were retching and asthenovegetative syndrome). Since June 2004, the patient increased administration of *B. oligonitrophilus* KU-1 (300 ml/day). According to ultrasonography data, size of metaplastic cancer in cellular tissue of pelvis minor decreased up to 30%. Then, the patient continued to receive *B. oligonitrophilus* KU-1 (50 ml/day, a week of administration changes by a week of interruption). In January 2005, the patient was hospitalized due to relapse of thrombophlebitis. Clot was operated. The patient received antithrombin drugs. Since January 2005, administration of *B. oligonitrophilus* KU-1 was stopped. As a result, there was a significant increase in CA 125 level in March 2005. In April 2005, administration of *B. oligonitrophilus* KU-1 was resumed (200 ml/day). State of health became significantly better until the middle of May 2005, when the patient died.

**Case 9** — Female, born in 1932. In 1993, high-grade differentiated breast adenocarcinoma was determined ($T_4N_0M_1$) with metastases into lymph nodes. Cancer resection was made at the Kharzizsk Oncological Center (Ukraine). In 2003, tumor relapse was determined (metastases in lymph nodes and mediastinum). Since December 2003, *B. oligonitrophilus* KU-1 was received (250 ml/day). In March 2004, a drastic increase in blood pressure was observed (until that time, the patient received clonidine as antihypertensive drug). Due to the above-mentioned reason, the administration of *B. oligonitrophilus* KU-1 was stopped. According to ultrasonography data, lymph nodes were not revealed in the mediastinum; subclavicular lymph nodes were also decreased. Since summer 2004, the patient had preventive administration of *B. oligonitrophilus* KU-1 (250 ml/day, a week of administration changes by a week of interruption). Along with *B. oligonitrophilus* KU-1, the patient received enalapril (due to high level of blood pressure). The patient died in May 2005.

**Case 10** — Male, born in 1946. In April 2003, gently differentiated adenocarcinoma of descending colon was revealed ($T_4N_2M_0$). In June 2003, the operation was made at Government State Service "Oncology" (Kazan, Russia). Since August 2003, the patient received *B. oligonitrophilus* KU-1 (100 ml/day). According to ultrasonography data (November 2003), there were metastases in the left lobe of liver (2.5 × 3.5 cm). Also, there were gangliform formations in both lobes of the liver (1–2 cm in diameter). Since November 2003, administration of *B. oligonitrophilus* KU-1 was increased up to 600 ml/day. Since December 2003, administration of *B. oligonitrophilus* KU-1 was decreased slightly (500 ml/day). In February 2004, administration of *B. oligonitrophilus* KU-1 was decreased up to 400 ml/day. According to ultrasonography data (April 2004), there were metastases (in the left lobe: 50 × 36 mm at $S_2$–$S_3$, 40 × 33 mm at $S_3$–$S_4$; in the right lobe: 27 × 25 mm at $S_6$–$S_7$) with fluid substances (10 mm in diameter), which are characteristic feature of undifferentiated tumor. Pancreas, spleen, and gall bladder were without pathology. In that period, the patient received 5-fluorouracil with leucovorin. Since April 2004, chemotherapy was absent; administration of *B. oligonitrophilus* KU-1 was increased up to 500 ml/day. Ultrasonography data (August 2004): liver metastases increased up to 7 cm in diameter, metastases in epicolon (until 15 mm in diameter), and retroperitoneal lymphatic glands were also revealed. In August 2004, the patient made cruise along Volga River. In the end of August, the patient worked at vegetable garden (he transported pails with potatoes). In September 2004, there was further decrease in state of health. Since September 2004, the supporting therapy (vicasol, prednisolone) was received. Since the end of September 2004, there was a drastic decrease in health state, quick development of jaundice and cachexy. Also, there was no appetite; insomnia and oedemata of legs were presented. The patient died in October 2004.

For comfort of data comparison, some key information is presented in Table 1. At present, there are three patients alive with rectal adenocarcinoma, thyroid adenocarcinoma, and *in situ* cancer. Their current survival periods are 7, 3, and 9 years, respectively. The patient with rectal adenocarcimoma (female, born in 1937) receives *B. oligonitrophilus* KU-1 regularly twice a year with prophylactic aim, while the two others have no restrain.

**TABLE 1**
**Some Key Information on Patients Under Study**

| Case # | Type of Tumor | Age at the Moment of Disease Detection | Daily Dose (ml) | Side Effects | Parallel Anticancer Treatment | Prophylactic Administration |
|---|---|---|---|---|---|---|
| 1 | Sigmoid colon tumor | 67 | 10–50 | Sicchasia | Absent | Absent |
| 2 | Breast adenocarcinoma | 67 | 400 | Absent | Absent | Absent |
| 3 | Ovary tumor | 42 | 200–700 | Absent | Topotecan | Absent |
| 4 | Rectosigmoid adenocarcinoma | 45 | 200–400 | Absent | Absent | Absent |
| 5 | Tumor of maxillary sinus | 60 | 50–400 | Blood pressure gain | Platinum-containing drugs and gamma irradiation | Absent |
| 6 | Metastatic backbone tumor | 76 | 200 | Absent | Absent | Absent |
| 7 | Stomach adenocarcinoma | 68 | 200 | Blood pressure gain | Absent | Absent |
| 8 | Double-sided ovary cystadenocarcinoma | 40 | 50–300 | Intracranial pressure gain | Cystatin | Present |
| 9 | Breast adenocarcinoma | 61 | 250 | Blood pressure gain | Absent | Absent |
| 10 | Adenocarcinoma of descending colon | 57 | 100–600 | Absent | 5-Fluorouracil plus leucovorin | Absent |

## DISCUSSION

The ability of some probiotic microorganisms to reduce the risk of cancer is a well-established phenomenon. It was shown that probiotics could reduce risk of cancer in the gastrointestinal tract[13,14] as well as at other sites of the human organism[15,16]; therefore, it is reasonable to suggest that probiotics have a systemic effect rather than a local one[17]. Although our results confirmed cancer-preventive properties of *B. oligonitrophilus* KU-1, the main finding is that peroral administration of *B.*

*oligonitrophilus* KU-1 may be a promising treatment approach. Assuredly, our research had a significant limitation because it was not double-blind or placebo-controlled. Also, computed tomography and magnetic resonance tomography data were absent for the majority of the studied patients. Four patients received conventional treatments in parallel. In all patients, however, deterioration in health state was detected after administration of cytostatic agents and radiotherapy. In this connection, it is interesting to note that parallel administration of *B. oligonitrophilus* KU-1 allowed us to downplay side effects of chemo- and radiotherapy, since hematological status was normalized owing to applied bacterium.

Fig. 3 demonstrates survival periods for the studied patients. It is clear from the figure that all patients demonstrated increased survival period in comparison with the standard prognosis given by the physician and presented in the literature[18]. Table 2 shows that the observed differences were significant. Concerning effectiveness of our method, the following remarks should be made. Of course, prognosis depends on various factors like type of cancer, stage of disease, age, and environmental factors. However, we consider that some approximation can be made. Namely, life prolongation is possible (approximately 12–18 months over standard prognosis) in patients with undifferentiated tumors. In patients older than 40 and younger than 60 with moderately differentiated tumors, elevation of survival over standard prognosis can reach 2 years. In patients with high-grade differentiated tumors and an absence of metastases into the vitally important organs (i.e., liver or lungs), life prolongation can reach 2–3 years over standard prognosis. Finally, we consider that total elimination of revealed metastases is possible in patients older than 60 (especially in case of breast cancer). Unfortunately, our method may be ineffective in children, especially with undifferentiated tumors. We also consider some recommendations important for a favorable outcome: a diet without preservatives, emulsifiers, synthetic dyes, smoked food, roast, marinades, pickles, or spirituous liquors; sour-milk diet, boiled veal meat and fish, boiled buckwheat, porridge, millet gruel, boiled rice, vegetables, and green tea are the most recommended eatables, while supercooling, superheating, isolation, psychic anxieties, and stress are contraindicated.

Finally, therapeutic efficiency, safety, and low price allow us to conclude on the principal utility of the suggested method. We hope that our results will be confirmed in double-blind, placebo-controlled clinical trials.

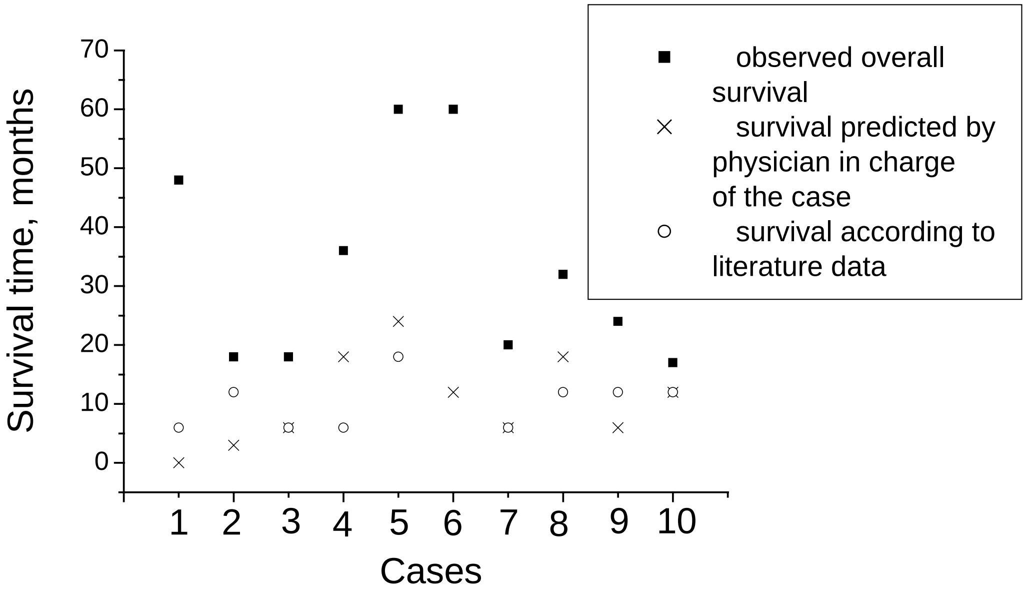

**FIGURE 3.** Survival period for patients that were under study and its comparison with predicted values.

**TABLE 2**
**Student's *t* test for Survival Period**

| Type of Comparison | t Value |
|---|---|
| Observed survival vs. survival predicted by physician in charge of the case | 4.67616 ($p < 0.01$) |
| Observed survival vs. survival according to literature data | 4.24492 ($p < 0.01$) |

## ACKNOWLEDGMENTS

We are very grateful to Dr. Anton N. Akulov from the Kazan Institute of Biochemistry and Biophysics (Kazan, Russia) for the scanning of MRIs. Also, we would like to thank Dr. Gyorgy Balazs (Semmelweis University, Department of Cardiovascular Surgery, Unit of CT & MRI, H-1122 Budapest, Határőr út 18, Hungary) and Dr. Miklos Garami (Semmelweis University, 2nd Department of Pediatrics, Unit of Pediatric Oncology, H-1094 Budapest, Tűzoltó u. 7-9, Hungary) for their kind help with the description of MRI results.

## BIOSKETCHES

**Sergey V. Malkov, PhD**, is Associate Professor in the Department of Genetics of the Kazan State University, Kazan, Russia. He is a developer of a new bacterial-based anticancer method.

**Vladimir V. Markelov, MD**, is a Vice-Director of Kazan Rehabilitation Medical Health Center “Sanatorium Krutushka”, Kazan, Russia. He has a strong commitment to the use of holistic principles in oncology. E-mail: krutushka_adm@mail.ru

**Gleb Y. Polozov, PhD**, is Assistant Professor in the Department of Genetics of the Kazan State University, Kazan, Russia. His main interest is isolation of soil microorganisms.

**Boris I. Barabanschikov, PhD, D.Sc**. is Professor, Head of Department of Genetics of the Kazan State University, Kazan, Russia. E-mail: borgenet@rambler.ru

**Alexander Y. Kozhevnikov MSc**, is a researcher in the Laboratory of plant genetics, Kazan Institute of Biochemistry and Biophysics, Kazan, Russia. His main interest is cultivation of soil microorganisms.

**Maxim V. Trushin, PhD,** is a postdoctorial fellow in the Laboratory of Pathogenesis, Kazan Institute of Biochemistry and Biophysics, Kazan, Russia. He is on the editorial board of several scientific journals and has published extensively in international journals on microbiology and intercellular communication. E-mail: mtrushin@mail.ru
